# The Jamaica asthma and allergies national prevalence survey: rationale and methods

**DOI:** 10.1186/1471-2288-10-29

**Published:** 2010-04-03

**Authors:** Eulalia K Kahwa, Novie O Younger, Yvonne B Wint, Norman K Waldron, Hermi H Hewitt, Jennifer M Knight-Madden, Kay A Bailey, Nancy C Edwards, Laurel R Talabere, Karen N Lewis-Bell

**Affiliations:** 1 The UWI School of Nursing, Mona, 9 Gibraltar Camp Way, University of the West Indies, Kingston 7, Jamaica; 2 Tropical Medicine Research Institute, University of the West Indies, Kingston 7, Jamaica; 3 Department of Obstetrics, Gynaecology and Child Health, University of the West Indies, Mona, Kingston 7, Jamaica; 4 School of Nursing and Department of Epidemiology and Community Medicine, Faculty of Health Sciences, University of Ottawa, 451 Smyth Road, Ottawa, Ontario, K1H 8 M5 Canada; 5 Department of Nursing, Capital University, 1 College and Main, Columbus, OH 43209-2394, USA; 6 Family Health, Ministry of Health, Jamaica, 2-4 King Street, Kingston 10, Jamaica

## Abstract

**Background:**

Asthma is a significant public health problem in the Caribbean. Prevalence surveys using standardized measures of asthma provide valid prevalence estimates to facilitate regional and international comparisons and monitoring of trends. This paper describes methods used in the Jamaica Asthma and Allergies National Prevalence Survey, challenges associated with this survey and strategies used to overcome these challenges.

**Methods/Design:**

An island wide, cross-sectional, community-based survey of asthma, asthma symptoms and allergies was done among adults and children using the European Community Respiratory Health Survey Questionnaire for adults and the International Study of Asthma and Allergies in Children. Stratified multi-stage cluster sampling was used to select 2, 163 adults aged 18 years and older and 2, 017 children aged 2-17 years for the survey. The Kish selection table was used to select one adult and one child per household. Data analysis accounted for sampling design and prevalence estimates were weighted to produce national estimates.

**Discussion:**

The Jamaica Asthma and Allergies National Prevalence Survey is the first population- based survey in the Caribbean to determine the prevalence of asthma and allergies both in adults and children using standardized methods. With response rates exceeding 80% in both groups, this approach facilitated cost-effective gathering of high quality asthma prevalence data that will facilitate international and regional comparison and monitoring of asthma prevalence trends. Another unique feature of this study was the partnership with the Ministry of Health in Jamaica, which ensured the collection of data relevant for decision-making to facilitate the uptake of research evidence. The findings of this study will provide important data on the burden of asthma and allergies in Jamaica and contribute to evidence-informed planning of comprehensive asthma management and education programs.

## Background

Asthma is one of the most common chronic respiratory diseases affecting adults and children worldwide [1]. Epidemiological studies indicate an increase in the prevalence of asthma and allergic disease in some countries and stability in the prevalence of asthma in other countries [2-4]. However, trend analyses of national asthma prevalence and international comparison of asthma prevalence in different populations have been constrained by differences in definitions and inconsistent measures of asthma used in prevalence surveys [5]. More recently, the development of standardized questionnaires such as the European Respiratory Health Survey (ECRHS [6] and the International Study of Asthma and Allergies in Children (ISAAC)[7] has facilitated common definitions and measures of asthma to enable international comparison of asthma prevalence within and across populations. To date, there have only been a handful of studies in a limited number of Caribbean countries that have used these standardized measures. These studies have primarily focused on children [8-11].

In 2007, the University of the West Indies, School of Nursing, Mona (UWISON) in collaboration with the Ministry of Health in Jamaica commissioned the Statistical Institute of Jamaica (STATIN) to carry out the Jamaica asthma and allergies national prevalence survey to quantify the asthma and allergies disease burden in the population using internationally standardized survey questionnaires.

The purpose of this paper is to describe methods used in the national prevalence survey of asthma, highlight the different steps followed in executing this survey, and discuss challenges associated with conducting such a survey in Jamaica and strategies used to overcome these challenges.

## Methods/Design

This island-wide, cross-sectional, community-based prevalence survey used a nationally representative sample of children aged 2 - 17 years and adults 18 years and older. Jamaica is a Caribbean island with a total population of approximately 2.6 million. The country is geographically divided into 14 parishes consisting of 5,235 enumeration districts (EDs) stratified into urban and rural EDs. There are 2,542 urban and 2,693 rural EDs.

Stratified multi-stage cluster sampling using EDs as primary sampling units (PSU) was done similar to other national surveys [12]. In the first stage of selection, a random sample of 342 EDs was selected with the probability of selection proportional to parish size. The sample was drawn independently from urban and rural EDs to ensure reliable estimates from both areas of Jamaica. The sampling frame used for the survey was a list of all EDs in each parish. Based on the 2001 Population Census [13], it was estimated that the selected EDs were comprised of approximately 3,719 dwellings across the 14 parishes of Jamaica (Table 1).

**Table 1:** Number of Enumeration Districts, Dwellings and Respondents selected by Parish

Parish	Jamaica		Children recruited			Adults recruited		
	
	No. of EDs	No. of Dwellings	No. of EDs	No. of Dwellings	Interviews Completed	No. of EDs	No. of Dwellings	Interviews Completed
Kingston	224	27204	16	200	138	10	110	151

St. Andrew	973	156137	62	538	340	56	448	349

St. Thomas	224	27301	16	200	74	10	110	86

Portland	187	23092	14	202	68	8	112	83

St. Catherine	906	128974	52	458	289	46	368	345

St. Mary	278	31403	18	234	92	12	144	103

St. Ann	319	43963	19	234	111	13	152	110

Trelawny	178	21263	14	210	115	8	210	108

St. James	347	48343	24	252	148	18	252	169

Hanover	159	19868	14	210	133	8	120	127

Westmoreland	322	41320	21	225	100	15	135	92

St. Elizabeth	308	40700	20	216	92	14	126	100

Manchester	343	50629	24	252	136	18	162	152

Clarendon	467	62843	28	288	181	22	198	188

**Total**	**5235**	**723040**	**342**	**3719**	**2017**	**258**	**2647**	**2163**

In the second stage, dwellings were selected from study EDs using systematic probability sampling which ensured that within study EDs each dwelling had the same probability of being selected. A list of all dwellings in each of the study EDs and a list of the names and addresses of each household head in these EDs was made using data from the 2002 Jamaica Labour Force Survey [14]. Selection of dwellings was done systematically starting from a random point determined by STATIN. A sample of 2,647 dwellings was selected to obtain the adult sample and an additional 1,072 dwellings were selected to obtain the sample for children bringing the total number of selected dwellings to 3,719. Table 1 shows the number of EDs, dwellings and respondents selected in each parish. Since the number of EDs selected was proportional to parish size, larger parishes had more EDs, dwellings and respondents. In some parishes, some dwellings had more than one household. Therefore the number of dwellings was smaller than the number of respondents.

The third stage of sampling involved the selection of individuals from private households. Institutionalised persons such as those in military camps, boarding schools, hospitals and prisons were excluded from the survey. Each household provided one adult and when possible one child. In households where there was no eligible child, only one adult was selected. In households where there were more than one eligible adult or child, the Kish selection table [15] was used to select one adult and one child for the survey. In order to use the Kish selection table, the interviewer listed serially all eligible adults and children who lived in the household on separate forms in order of decreasing age starting with the oldest male to the youngest male and then the oldest female to the youngest female. The Kish selection table had rows defined by the last digit on the questionnaire number. Columns were defined by the possible number of eligible persons in the household, while each cell had a number (*k*) to indicate the participant to be selected from the household list. The number (*k*) in the cell corresponding to the number of eligible adults or children in the household on the interviewer's list and the last digit on the questionnaire number indicated the *k*^th ^individual on the interviewer's list who was invited to participate in the study. This selection strategy yielded a total sample of 2,163 adults and 2, 017 children. The response rate was 89% among adults and over 80% among children.

### Eligibility criteria

Children aged 2-17 years and adults aged 18 years and older who were residents in the selected households were eligible to participate in the survey. Individuals were considered resident in a household if they slept in that household at least three nights per week [12]. Adults diagnosed with diseases that might mimic asthma such as chronic obstructive pulmonary disease (COPD), chronic bronchitis or emphysema, congestive heart failure, and pulmonary embolism were excluded from the survey. Persons on beta blockers were also excluded as these drugs are often associated with wheezing [16]. Children under the age of 2 years were excluded from the survey since there are other conditions such as bronchiolitis and other causes of wheezing which could impact the accuracy of the asthma diagnosis below this age. Coughing and wheezing are symptoms of bronchiolitis which can be difficult to distinguish from wheezing and coughing due to asthma [16].

### Survey questionnaires

The ECRHS (Phase 2) [6] and the ISAAC (Phase 2) Questionnaires [7] were used to assess the prevalence of asthma in adults and children respectively. Both questionnaires were administered in English.

### Interviewer training

Training sessions were held for interviewers and staff members of STATIN prior to the start of data collection activities. Interviewers and members of STATIN's permanent staff participated in a three-day training before the pre-test of questions on both the ECRHS and the ISAAC questionnaires. Another four-day training session was held to train interviewers and supervisors before the main survey to ensure consistency among interviewers. The intent of each question was carefully reviewed. In addition, interviewers practiced administering questionnaires, obtaining informed consent and assent from relevant participants, and using the Kish selection table to select eligible participants within households.

### Pre-testing of questionnaires

Both the ISAAC and ECRHS questionnaires were pre-tested using a sample of households from various parishes. The main objectives of the pre-test were to assess the wording, sequence and clarity of questions and instructions in the questionnaire and to estimate the average duration of an interview. For the pre-test, interviewers were assigned to areas of the island which, because of environmental factors, are perceived to have high prevalence of asthma. Each interviewer interviewed a convenience sample of one adult with asthma, one adult without asthma, two children with asthma and one child without asthma. Households used in the pre-test were not included in the main survey.

Feedback from the pre-test was used to make minor cultural modifications in the questionnaire. The wording of the question asking "Have you ever had asthma" on the ECRHS questionnaire and the question "Has your child ever had asthma" on the ISAAC questionnaire were modified to "Have you ever had asthma or even a touch of asthma?" and "Has your child ever had asthma or even a touch of asthma?" respectively. These amendments were based on the local terminology used to describe individuals with mild or moderate asthma [17]. On the ECRHS questionnaire, an item eliciting the "number of ganja (marijuana) spliffs smoked per day" was added to questions on smoking. In order to assess the socioeconomic status (SES) of participants, items on indices relevant to Jamaica were added to both questionnaires. In addition to education and occupation, information was gathered on household belongings, crowding, type of housing [18] and household income. Questions were also added on the cost of asthma medication in the last 12 months and whether or not the participants had health insurance. These changes were made in consultation with key stakeholders from the Ministry of Health in Jamaica.

### Questionnaire reliability

The test - retest reliability of questionnaire items was assessed. During the pre-test each respondent was interviewed twice and the second interview was administered five days after the first interview. Kappa statistics were the primary means used to assess the reliability of responses. Table 2 shows the test-retest reliability for responses to items eliciting identifying information. For all information except dwelling and constituency, the reliability as defined by the kappa statistics exceeded 60%. For variables that were not in numeric form, a percentage agreement was estimated because these variables tended to have more than five categories and therefore there were very few responses within each category. For these variables, the percentage agreement exceeded 70%. Where they could be estimated, kappa statistics ranged from 63% to 100% for nearly all items aimed at classifying asthma symptoms. However, kappa statistics for items eliciting information on the frequency of symptoms were low, 25% for frequency of shortness of breath and 11.8% for timing of worsening asthma symptoms during the menstrual cycle. The low values could be due to variability in asthma symptoms [19].

**Table 2:** Kappa statistics and observed percentage agreement for the test-retest responses to respective questionnaire items

Variable	Observed agreement (%)	Kappa Statistic (%)
Identifying Information		
Parish	75.0	69.8***

Region	90.0	62.6***

Dwelling	90.0	-5.3

Constituency	65.0	30.0**

Household	95.0	64.3**

Enumeration District	90.0	62.6***

Interviewer No.	95.0	92.1***

Interviewer's name	75.0	-

Asthma Symptoms Have you had wheezing or whistling in your chest any time in the last 12 months	95.0	90.0

Have you been at all breathless when the wheezing noise was present	100.0	100.0

Have you had this wheezing or whistling when you did not have a cold	90.0	73.4

Have you woken up with a feeling of tightness in your chest at any time in the last 12 months	95.0	89.8

Have you had an attack of shortness of breath that came on during the day when you were at rest at anytime in the last 12 months	100.0	100.0

Have you had shortness of breath that came on following strenuous activity at anytime in the last 12 months	100.0	100.0

Have you been woken up by an attack of shortness of breath at anytime in the last 12 months	80.0	63.1

How many times a week on average have you been woken by shortness of breath in the last 3 months?	33.0	25.0

Have you ever noticed that you had respiratory symptoms at a particular time in your monthly cycle	42.9	22.8

Have you ever noticed that your asthma got worse with your monthly cycle	40.0	11.8

* < 0.05, ** < 0.01, *** < 0.001)

### Project administration

The project was administered by the UWI School of Nursing, Mona (UWISON). The research team comprised of pulmonologists, allergists, nurse scientists, epidemiologists, and a biostatistician was responsible for the study design and data management. STATIN was contracted to conduct the survey. A project implementation committee consisting of members of the research team from the University of the West Indies, representatives from the Ministry of Health in Jamaica and STATIN, was established to plan and oversee project activities. The project implementation committee held regular meetings to review the progress of project activities. Prior to the start of data collection, the committee reviewed and approved cultural modifications to the questionnaire. A project coordinator from UWISON had overall responsibility for coordinating all project activities. Fieldwork supervisors were assigned to oversee the work of interviewers. Other specific duties of supervisors included reviewing the starting point and boundaries of EDs with each interviewer, checking correct entry of all data on questionnaires and taking corrective action as required. Supervisors also prepared weekly data collection logs and progress reports on data collection activities.

### Ethical considerations

Ethical approval was granted by the Ethics Committees of the UWI/University Hospital of the West Indies/Faculty of Medical Sciences and the Ministry of Health, Jamaica. All respondents selected for the survey were required to sign a consent form before interviews were conducted. A copy of the consent form was given to respondents. Adults were required to give informed consent for their children to participate in the survey. Children aged 8 - 17 years were also required to sign an assent form to show that they had agreed to participate in the survey. Information for children under age 12 years was obtained from their caregivers. Children aged 13 years and older were interviewed in the presence of their caregivers. Individuals who were under 18 years old but were responsible for themselves, married or with children (emancipated minors) consented for themselves.

### Data management and Data Quality

Census and Survey Processing System (CSPro) software was used for entering, editing and tabulating survey data. This interactive user-friendly program is capable of performing checks for consistency, range and skip patterns and its use enhances data quality by ensuring that data are entered only into predetermined fields. When error messages were displayed on the screen because logic was in the wrong place or there was incorrect syntax or spelling of commands, CSPro provided instructions on how to proceed. At the end of data entry, an edit program was run which indicated whether or not data compilation was successful.

### Data analysis

Data analysis was done using algorithms in Stata version 9.0 appropriate for handling survey data and incorporating sampling weights. In order to ensure valid estimates of variability that incorporated clustering within PSUs, EDs with fewer than three participants were combined with a neighbouring ED so that at least three persons were in the newly formed PSU. The number of EDs per parish and the average number of households per ED were used to obtain the probability of selecting an ED and a household, respectively. The inverse of the product of these probabilities produced the raw sample design weights, which were further multiplied by post-stratification weights to yield sampling weights indicating an approximate number of persons in the population represented by each participant. Application of weights to parameter estimation was intended to minimise the effects of discrepancies between the population and sample distributions. Post-stratification adjustment cells were defined by the parish, sex and age distribution of respondents according to census data provided by STATIN [20]. Thus, weighted estimates produced by data analysis accounted for the multistage sampling design as well as any sampling frame inadequacies and were considered nationally representative. Numeric summaries including prevalence estimates, relative odds for correlates of asthma prevalence and other estimates of population parameters were obtained.

### Recruitment

A total of 2,163 adults consisting of 936 (43.3%) males and 1,227 (56.7%) females were recruited. The ages of adult participants ranged from 18 - 92 years, with the majority of adult participants, 67.6%, being in the 18 - 44 years age group. Those 65 years and older accounted for 10.2% of the sample. A total of 1,018(47%) adult participants were from urban and 1,145(53%) were from rural parishes.

A total of 2,017 children aged 2-17 years were recruited consisting of 1,019 (50.5%) males and 998 (49.5%) females. Over 60% of the children were in the 5-14 years age group and just fewer than 20%, of children were in the 2-4 years age group. In the sample of children 912 (45.2%) were from urban and 1,105 (55%) from rural parishes.

Tables 3 and 4 show the parish by sex distribution of adults and children recruited for the survey. The sampling of EDs proportionate to parish size is reflected in larger parishes such as St. Andrew and St. Catherine which have larger numbers of participants. Tables 5 and 6 show weighted and unweighted age by sex distribution of the adult and children samples as well as the distribution in the Jamaican population. For the adult sample, the male to female distribution was 48.3% males and 51.6% females in the Jamaican population versus 43.3% males and 56.7% females in the sample. For the 2-17 years age group, the distribution was 50.6% males and 49.3% females in the Jamaican population versus 50.5% males and 49.5% females in the sample. The male to female distribution in the sample of children was closer to the population distribution. Population data used were those obtained from the 2001 census [13].

**Table 3:** The Unweighted Distribution of the Adult Sample by Sex and Parish of Residence (n = 2,163)

Parish	Males	Females	Total
	
	n (%)	n (%)	n (%)
Kingston	70 (7.5)	81 (6.6)	151 (7.0)

St. Andrew	135 (14.4)	214 (17.5)	349 (16.1)

St. Thomas	35 (3.8)	51 (4.2)	86 (4.0)

St. Catherine	134 (14.3)	211 (17.2)	345 (16.0)

Portland	40 (4.3)	43 (3.5)	8 (3.8)

St. Mary	51 (5.4)	52 (4.2)	103 (4.8)

St. Ann	58 (6.2)	52 (4.2)	110 (5.0)

Trelawny	36 (3.8)	72 (5.9)	108 (4.9)

St. James	77 (8.2)	92 (7.5)	169 (7.8)

Hanover	44 (4.7)	83 (6.7)	127 (6.0)

Westmoreland	46 (4.9)	46 (3.8)	92 (4.3)

St. Elizabeth	52 (5.6)	48 (3.9)	100 (4.6)

Manchester	66 (7.1)	86 (7.0)	152 (7.0)

Clarendon	92 (9.8)	96 (7.8)	188 (8.7)

**Total**	**936 (43.3)**	**1227 (56.7)**	**2163 (100)**

**Table 4:** The Unweighted Distribution of Children in the Sample by Sex and Parish of Residence (n = 2,017)

Parish	Males	Females	Total
	
	n (%)	n (%)	n (%)
Kingston	65 (6.4)	73 (7.3)	138 (6.8)

St. Andrew	164 (16.1)	176 (17.6)	340 (16.9)

St. Thomas	37 (3.6)	37 (3.7)	74 (3.7)

St. Catherine	142 (13.9)	147 (14.7)	289 (14.3)

Portland	40 (3.9)	28 (2.8)	68 (3.4)

St. Mary	56 (5.5)	36 (3.6)	92 (4.6)

St. Ann	58 (5.7)	53 (5.3)	111 (5.5)

Trelawny	63 (6.2)	52 (5.2)	115 (5.7)

St. James	74 (7.3)	74 (7.4)	148 (7.3)

Hanover	58 (5.7)	75 (7.5)	133 (6.6)

Westmoreland	51 (5.0)	49 (4.9)	100 (5.0)

St. Elizabeth	49 (4.8)	43 (4.3)	92 (4.6)

Manchester	78 (7.7)	58 (5.8)	136 (6.7)

Clarendon	84 (8.2)	97 (9.7)	181 (9.0)

**Total**	**1019 (50.5)**	**998 (49.5)**	**2017 (100.0)**

**Table 5:** Age and Sex Distribution (Actual & Weighted) of adult sample and the Jamaican Population

	Sample						Jamaican population		
	**Males**		**Females**		**Total**		**Males**	**Females**	**Total**

	**Unwtd**	**Wtd**	**Unwtd**	**Wtd**	**Unwtd**	**Wtd**			

	**n(%)**	**%**	**n (%)**	**%**	**n (%)**	**%**	**n (%)**	**n (%)**	**n (%)**

18-24	128 (13.7)	20.5	173 (14.1)	20.3	301 (13.9)	20.4	154602 (19.8)	160551 (19.3)	315153 (19.6)
25-34	208 (22.2)	24.8	323 (26.3)	26.3	531 (24.6)	25.6	193240 (24.8)	211237 (25.4)	404477 (25.1)
35-44	237 (25.3)	21.3	341 (27.8)	21.9	578 (26.7)	21.6	163930 (21.1)	176453 (21.2)	340383 (21.1)
45-54	158 (16.9)	13.9	180 (14.7)	13.1	338 (15.6)	13.5	105526 (13.5)	105414 (12.6)	210940 (13.1)
55-64	92 (9.8)	9.2	100 (8.2)	8.1	192 (8.9)	8.6	70473 (9.0)	70455 (8.5)	140928 (8.7)
65-74	68 (7.3)	6.9	55 (4.5)	6.1	123 (5.7)	6.4	53757 (6.9)	58214 (6.9)	111971 (6.9)
75-84	35 (3.7)	2.7	44 (3.6)	3.1	79 (3.7)	2.9	28015 (3.5)	35126 (4.2)	63141 (3.9)
≥ 85	10 (1.1)	0.7	11 (0.9)	1.0	21 (1.0)	0.9	9219 (1.1)	15148 (1.8)	24367 (1.5)

**Total**	**936**		**1227**		**2163**		**778762**	**832598**	**1611360**

**Table 6:** Age and Sex Distribution (Actual & Weighted) of the children sample and the Jamaican Population

Sample							Jamaica population		
		**Males**	**Females**		**Total**		**Males**	**Females**	**Total**

	**Unwtd**	**Wtd**	**Uwtd**	**Wtd**	**Unwtd**	**Wtd**			

	**n (%)**	**%**	**n (%)**	**%**	**n (%)**	**%**	**n (%)**	**n (%)**	**n (%)**

2-4	189 18.6)	18.6	181 (18.1)	18.4	370 (18.3)	18.5	83348 (18.6)	80342 (18.4)	163690 (18.5)
5-9	343 (33.7)	33.4	323 (32.4)	33.2	666 (33.0)	33.3	149653 (33.4)	145219 (33.2)	294872 (33.3)
10-14	317 (31.1)	31.1	322 (32.3)	31.2	639 (31.7)	31.2	139372 (31.1)	136507 (31.2)	275879 (31.2)
15-17	170 (16.7)	16.9	172 (17.2)	17.2	342 (16.9)	17.1	75878 (16.9)	75307 (17.2)	151185 (17.1)

**Total**	**1019**		**998**		**2017**		**448251**	**437375**	**885626**

## Discussion

To our knowledge, this is the first population based survey in the Caribbean to determine the prevalence of asthma and allergies both in adults and children using standardized methods. Previous asthma prevalence studies in the Caribbean using standardized questionnaires focused mainly on asthma in children [9-11]. The main strengths of this study include: 1) the use of survey methods appropriate for national health surveys, 2) the use of standardised methods consistent with current thinking on measuring asthma in prevalence studies, and 3) the use of a nationally representative sample covering both rural and urban areas. Response rates exceeded 80% enabling the acquisition of credible national asthma prevalence estimates in both adults and children. This approach facilitated cost-effective gathering of high quality asthma prevalence data that will enable international and regional comparison. Another unique feature of this study was partnership with the Ministry of Health in Jamaica which ensured the collection of data relevant for decision-making facilitating the uptake of research evidence. A technical report summarizing the findings of this study was submitted to the Ministry of Health.

Collection of survey data can be expensive and time consuming. In order to reduce time and expense involved in gathering data in large surveys, cluster sampling is often used [7,21,22]. However, cluster sampling usually results in a lack of independence of observations obtained from units within the same cluster [23]. To obtain valid estimates of variability, analyses of data from surveys that use cluster sampling should account for these correlated data as well as the multistage sampling design [23,24]. In this study, data analysis accounted for the complex survey design, thereby yielding parameter and variability estimates that would allow for valid inferences about the fixed population that was sampled.

While the sample used for the survey was representative, there were slight differences in the sex distribution between the adult sample (43.3% males and 56.7% females) and the Jamaican population (48.3% males and 51.6% females). However, this discrepancy would not adversely affect parameter estimates since application of sampling weights to data analysis is aimed at minimising the effects of discrepancies between the population and sample distributions. As illustrated in Table 5, applying sampling weights to the estimation of frequency distribution resulted in percentages that were closer to the values obtained for the population. The tendency to recruit more females than males has also been observed in other national surveys in Jamaica [25] and elsewhere [26]. In Jamaica, this variance could be attributed to the fact that some men fail to meet the eligibility criteria of sleeping in the home for at least three nights per week (12). The male/female distribution in the children sample closely reflected the male/female distribution in the population.

There were minimal logistical challenges during the survey due to STATIN's experience with national surveys and knowledge of the terrain. However, in a difficult setting such as Jamaica, interviewers occasionally had to halt data collection activities temporarily due to violence in some urban communities and wait until community leaders told them when it was safe to re-enter communities. These occurrences resulted in a delay of one month in completing the survey. Also for security reasons resulting from violent activities in some urban areas, gated communities now common in Jamaica, were sometimes difficult to access by interviewers. This problem was resolved by establishing relationships with strata managers in order to gain access to these communities. Establishing a good relationship with community leaders also ensured security for interviewers.

## Conclusion

Asthma prevalence data obtained through the use of standardized questionnaires are critical to facilitate international comparison of prevalence data in populations and monitoring national and international asthma prevalence trends. The Jamaica Asthma and Allergies National Prevalence Study utilized methods appropriate for the execution of national health surveys. The findings of this study will provide important data on the burden of asthma and allergies in Jamaica inform policy decisions on asthma and contribute to evidence-based planning of comprehensive asthma management and education programs.

## Competing interests

The authors declare that they have no competing interests.

## Authors' contributions

EK was the principal investigator; in addition NY, NW, YW, LT and HH were responsible for proposal development and obtaining grant funds. NY and EK were responsible for data management and analysis. YW, HH, LT, JKM, NE, KB and NW were responsible for data interpretation. EK and NY drafted the original manuscript. All authors reviewed the manuscript and contributed to the intellectual content of the paper.

## Pre-publication history

The pre-publication history for this paper can be accessed here:

http://www.biomedcentral.com/1471-2288/10/29/prepub
